# Vitreous humor in the forensic toxicology of quetiapine and its metabolites

**DOI:** 10.1007/s11419-024-00687-z

**Published:** 2024-04-14

**Authors:** Danai Moschovakou, Stamatina-Panagoula Ntoupa, Artemisia Dona, Sotirios Athanaselis, Chara Spiliopoulou, Panagiota Nikolaou, Ioannis Papoutsis

**Affiliations:** 1 Department of Forensic Medicine and Toxicology, School of Medicine, National and Kapodistrian University of Athens, 75 Mikras Asias, 115 27 Athens, Greece; 2 Laboratory of Pharmaceutical Analysis, Department of Pharmacy, National and Kapodistrian University of Athens, 157 71 Athens, Greece

**Keywords:** Quetiapine, Vitreous humor, Forensic toxicology, GC/MS

## Abstract

**Purpose:**

Τhe aim of the present study was to investigate the use of vitreous humor as an alternative biological material in forensic toxicology for the determination of quetiapine, 7-hydroxy-quetiapine, and nor-quetiapine. The distribution of these substances in vitreous humor was studied by determining and correlating their concentrations in vitreous humor with the respective concentrations in blood.

**Methods:**

During this study, a method for the determination of these substances was developed, validated and applied to postmortem samples obtained from 16 relative forensic cases. The sample preparation procedure included the isolation of the analytes from vitreous humor and blood samples using solid-phase extraction, with Bond Elut LRC C18 columns followed by derivatization with BSTFA with 1% TMCS prior to GC/MS analysis.

**Results:**

The developed method is characterized by a dynamic range of 10.0–1000.0 ng/mL (*R*^2^ ≥ 0.991) for the three substances, with a limit of detection and quantification of 3.0 and 10.0 ng/mL, respectively. Accuracy and precision were below 8.09% and 8.99%, respectively, for both biological materials, while absolute recovery for the three substances was greater than 81%. According to the results, quetiapine, 7-hydroxy-quetiapine, and nor-quetiapine are easily distributed in vitreous humor.

**Conclusion:**

The results of the study indicate the usefulness of vitreous humor in toxicological analysis for the determination of these substances, especially when the traditional biological materials are not available. The levels of quetiapine and its metabolites in vitreous humor as well as the vitreous humor to blood concentration ratios can provide important information for a more thorough toxicological investigation of forensic cases.

## Introduction

The most frequently used biological samples in toxicological analysis are blood and urine. However, it is common these classical biological materials to be absent during the investigation of forensic cases [1]. In addition, when the postmortem interval (PMI) is long, the concentration of many substances of interest in postmortem blood might be higher than in blood at the time of death, due to their postmortem redistribution [2]. Therefore, the use of alternative biological samples is studied, as they are likely to provide important information for a more thorough toxicological investigation of forensic cases including drug overdose. Vitreous humor is a transparent gelatinous matrix composed of water (99%) and fibrillar proteins (collagen and hyaluronic acid) [3]. The utility of this alternative postmortem biological sample in toxicological analysis has been studied in recent years, especially when blood and/or urine samples are not available or appropriate for reliable analysis [4]. Many drugs and other substances are distributed from blood to vitreous humor through blood–retina barrier (BRB) [5]. The physicochemical properties and pharmacokinetics of the substances, as well as the condition of BRB and the eye affect this permeation [6].

The utility of vitreous humor in forensic toxicology is highlighted by its anatomical advantages during its sampling [3, 7], and its purity (low percentage of proteins and other macromolecules) makes it suitable for qualitative analysis of many drugs as their protein binding in this biological fluid is limited or none [8]. As an isolated material, it is less affected by putrefaction and redistribution than other parts of the body [7]. Therefore, it is an important biological matrix for the determination of several substances during toxicological analysis. The distribution of some categories of substances like antidepressants [9], benzodiazepines [10], psychotropic drugs, and drugs of abuse, such as opiates [11], amphetamines [12], cocaine, and its metabolites [13] to vitreous humor has already been studied.

Quetiapine is a second-generation antipsychotic drug that is commercially available as quetiapine fumarate, mainly by AstraZeneca as Seroquel*®* [14], and is used primarily on schizophrenia, bipolar disorder, and other psychotic disorders [15, 16]. There is also evidence that quetiapine may benefit patients diagnosed with a mental illness who are also dependent on cocaine [17]. During the last years, quetiapine has been associated with case reports of patient misuse and abuse and there are indications that it possesses an addictive potential [18]. Quetiapine is rapidly absorbed after *per os* administration and it is extensively metabolized mainly to nor-quetiapine (*N*-desalkyl-quetiapine) and 7-hydroxy-quetiapine, which are detected at notable levels in body fluids after quetiapine administration [19].

Τhe aim of the present study was to investigate the use of vitreous humor as an alternative biological matrix in forensic toxicology for the determination of quetiapine and its main two metabolites. The distribution of these substances in vitreous humor should be studied, by correlating their concentrations in vitreous humor with the respective ones in blood samples. To the best of our knowledge, only a few methods have been reported for the determination of quetiapine in vitreous humor [20, 21] using either liquid chromatography (LC) with UV–DAD detector [20] or gas chromatography–mass spectrometry (GC/MS) [21], whereas there are several published methods applied to blood samples using mainly LC/MS/MS [22–25] and less GC/MS [26]. While LC techniques are mostly preferred due to simpler sample preparation and lack of derivatization requirements, GC/MS remains a widely used technique available in most forensic laboratories. For this purpose, a GC/MS method was developed, optimized and validated for the simultaneous determination of quetiapine and its main metabolites, 7-hydroxy-quetiapine and nor-quetiapine, in blood and vitreous humor samples. Only few studies concerning the determination of quetiapine in vitreous humor exist [20, 21, 27], while no study has been performed for the distribution of the main metabolites of quetiapine in this material. The developed method was applied to postmortem samples obtained from 16 cases, in which the studied substances were detected during routine toxicological analysis, or quetiapine was one of the prescribed drugs of the deceased.

## Materials and methods

### Chemicals and reagents

Reference standard solutions of quetiapine, *N*-desalkyl-quetiapine, 7-hydroxy-quetiapine, and quetiapine-*d*_*8*_ (Internal Standard, I.S.) at a concentration of 1.00 mg/mL (> 99.9% pure), were purchased from LGC Promochem (Molsheim, France). All standards were stored according to the instructions of their commercial certificates.

The solvents used (methanol, acetonitrile, dichloromethane and isopropanol) were of high-performance liquid chromatography (HPLC) grade and were purchased from Merck (Darmstadt, Germany). Analytical reagents were purchased as follows: *N*,*O*-bis(trimethylsilyl)-trifluoracetamide (BSTFA) with 1% trimethylchlorsilane (TMCS) from Sigma-Aldrich (Steinheim, Germany), analytical grade ammonium hydroxide (NH_4_OH), and sodium-dihydrogen phosphate dehydrate (NaH_2_PO_4_.2H_2_O) were obtained from Merck (Darmstadt, Germany). Bond Elut LRC C18 (Sorbent Mass 200 mg, Column Volume 10 mL) columns that were used for solid-phase extraction (SPE) were obtained from Agilent Technologies (Santa Clara, CA, USA).

Drug-free blood and vitreous humor samples were obtained from forensic cases and after their verification as negative for drugs by GC−MS analysis, before their use, were pooled.

### Calibrators and quality control samples

Six working standard solutions containing quetiapine, *N*-desalkyl-quetiapine, 7-hydroxy-quetiapine, at the following concentrations 0.20, 0.40, 1.00, 3.00, 10.0, and 20.0 μg/mL, were prepared by mixing the appropriate volumes of the corresponding stock solutions of each compound and then by diluting with acetonitrile. Spiked blood or vitreous humor samples for calibration curves (calibrators) were prepared by spiking 950 μL of blank human blood or vitreous humor with 50 μL of the mixed working standard solutions. The six calibrators contained all three analytes of interest at concentrations of 10.0, 20.0, 50.0, 150, 500, and 1000 ng/mL.

Additional working standard solutions containing quetiapine and its two main metabolites were prepared (at three different concentrations 0.60, 2.00 and 16.00 μg/mL) from different stock solutions than the ones used for calibrators, to prepare blood or vitreous humor quality control (QC) samples. The three QC samples contained 30.0, 100 and 800 ng/mL of all three analytes of interest, and were prepared in a similar way with the one of the calibrators.

A working internal standard solution containing quetiapine-*d*_*8*_ at 1.00 μg/mL was prepared by diluting the appropriate volume of the corresponding stock solution with acetonitrile.

Calibration curves (based on the peak area ratio of each analyte to the internal standard) were plotted over 4 days and used to calculate analyte concentrations.

### GC−MS analysis and apparatus

**Fig. 1:**
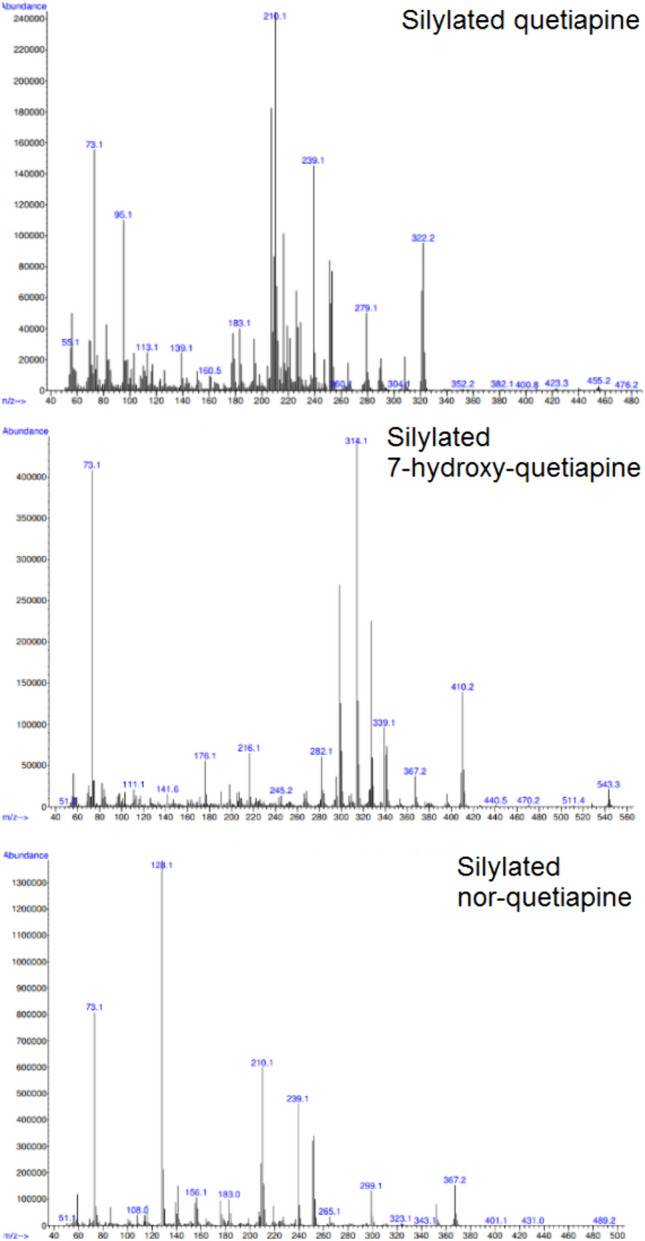
The mass spectra of the three silylated analytes of interest

The chromatographic analysis of quetiapine and its metabolites was performed on an Agilent GC−MSD model 6890N/5975 equipped with a DB-5MS fused silica column (30 m × 0.25 mm i.d. × 0.25 μm film thickness) supplied by Agilent Technologies (IL, USA). Helium was used as the carrier gas at a flow rate of 1.0 mL/min. 1 μL was injected in the splitless mode using an Agilent 7683B Series autosampler system. The optimized GC conditions were as follows: initial column temperature of 100 °C was held for 1 min, and then was increased to 300 °C at a rate of 30 °C/min, where it was held for 13 min. Injector, ion source, and interface temperatures were set at 260, 230 and 280 °C, respectively. The mass spectrometer was operated in electron impact (EI) ionization with selective ion monitoring (SIM) mode. The mass fragments used for the identification of the analytes were: *m/z* **322**, 251, and 279 for silylated quetiapine, *m/z* **314**, 298, and 327 for silylated 7-hydroxy-quetiapine, and *m/z* **128**, 210, and 252 for silylated nor-quetiapine. The bold marked ions were used for the quantification of the analytes. The respective mass fragment of the internal standard was **330** (quetiapine-*d*_*8*_). The mass spectra of the three silylated analytes of interest are presented in Fig. 1.

An MT 19 vortex (Chiltern, London, UK) was used for the mixing of standards and samples during their preparation. A 691 digital pH-meter (Metrohm, Herisau, Switzerland) with a glass electrode was used for pH adjustments. An SPE manifold HyperSep Thermo Scientific (Thermo Fischer Scientific, MA, USA) was used during sample preparation step. An evaporating unit connected with nitrogen (Reacti-Vap PIERCE Model 18780, Rockford, IL, USA) used during the sample preparation step. Centrifugation was performed with a Sigma 4K10 centrifuge (Osterade, Germany).

### Sample preparation

In all samples (1.0 mL of blood or vitreous humor), 50 μL of the working internal standard solution was added and the samples were vortex mixed for 15 s. Therefore, all calibrators, QC and casework samples contained 50.0 ng/mL of quetiapine-*d*_*8*_. Then, a volume of 4 mL of phosphate buffer *pH* 7.0 was added and vortexed. Centrifugation was performed for 10 min at 3000 rpm. The supernatants were then loaded on SPE cartridges that were previously conditioned with 2 mL methanol and 2 mL phosphate buffer *pH* 7.0. The samples were slowly passed (under low vacuum) through the cartridge at a rate of 1 mL/min and then the columns were washed with 2 mL distilled water. A 600 μL volume of *n*-hexane was then added, and columns were vacuumed for 10 min (> 10 mmHg). Finally, the analytes were eluted twice with 1.5 mL freshly prepared mixture solution of dichloromethane: isopropanol: ammonia (85:15:2, *v*/*v*/*v*). The eluates were then evaporated to dryness under continuous N_2_ stream. 50 μL of acetonitrile were used to reconstitute the dried residues, while 50 μL of BSTFA with 1% TMCS was added for the derivatization of the analytes. Derivatization was performed in sealed tubes at 70 °C for 30 min. After cooling the tubes, the derivatized extracts were transferred to glass vials and 1 μL was injected into the GC–MS system (splitless mode).

### Method validation

The validation of the developed method followed international guidelines [28, 29] and the parameters such as selectivity, specificity, linearity, sensitivity, precision, accuracy, recovery, and matrix effect were evaluated.

Selectivity of the method was examined by analyzing six different blank vitreous humor and blood samples, in which the absence of the analytes was confirmed.

Specificity of the developed method was studied by analyzing six spiked vitreous humor and blood samples containing substances, at a concentration of 500 ng/mL, that are commonly detected in quetiapine-related forensic cases. Substances such as paracetamol, diazepam, nordazepam, alprazolam, bromazepam, 7-amino-flunitrazepam, lorazepam, phenobarbital, olanzapine, chlorpromazine, levomepromazine, biperiden, zolpidem, tramadol, amphetamine, methamphetamine, 3,4-methylenedioxymethamphetamine, lidocaine, ketamine, nor-ketamine, ephedrine, D9-tetrahydrocannabinol, 11-nor-9-carboxy-D9-tetrahydrocannabinol, buprenorphine, norbuprenorphine, methadone, morphine, codeine, 6-monoacetylmorphine, cocaine, benzoylecgonine, and ecgonine methyl ester were examined to determine the possible interferences of exogenous compounds.

Sensitivity of the method was evaluated by defining the LOD and LOQ for each of the studied compounds. For both vitreous humor and blood, spiked samples at low concentrations were analyzed, to determine the lowest level which corresponds to a signal-to-noise ratio equal or higher than 3 and equal or higher than 10, for LOD and LOQ, respectively.

Calibration curves were obtained on four different days for blood and vitreous humor samples to determine linearity. Concentration values were 10.0, 20.0, 50.0, 150.0, 500.0, 1000.0 ng/mL for each analyte with I.S. concentration at 50 ng/mL. The peak area ratio of each studied compound to the area of the IS, against the corresponding concentration was used for the quantification of the samples. Following calibration curves’ assembly, regression lines ($y=ax+b$, where $x$ was each analyte’s concentration, and $y$ the respective peak area ratio) were calculated, according to the least squares regression analysis with a weighing factor of $1/{x}^{2}$ due to the wide range of concentrations of each analyte.

Precision (*% RSD*) and accuracy (*% E*_*r*_, mean relative error) of the method were determined by intra-day and inter-day analyses. Intra-day precision and accuracy were studied by analyzing six quality control (QC) vitreous humor and blood samples of the three concentration levels; low (30.0 ng/mL), intermediate (100.0 ng/mL), and high (800.0 ng/mL). Inter-day assessment was examined by analyzing the same concentration levels of QC vitreous humor and blood samples in 4 days.

Absolute recovery and matrix effect were determined at the three QC concentration levels by analyzing six blood and vitreous humor samples spiked before the extraction procedure (Set 1), six blood and vitreous humor samples spiked after the extraction procedure (Set 2), and six mixed standard solutions of the studied substances in acetonitrile (Set 3) at each concentration level. For each analyte, absolute recovery was calculated by dividing the mean peak area of analytes obtained in Set 1 (before extraction) to those in Set 3 multiplied by 100, while matrix effect was obtained by dividing the mean peak area of analytes obtained in Set 2 (after extraction) to those in Set 3 multiplied by 100.

## Results

The validation of the developed method for quetiapine, 7-hydroxy-quetiapine and nor-quetiapine was fully acceptable in both biological samples and the results are presented in detail below. During the examination of method selectivity and specificity, no interferences by any endogenous and exogenous compounds were observed at the retention time of each analyte of interest in both biological samples.

**Fig. 2:**
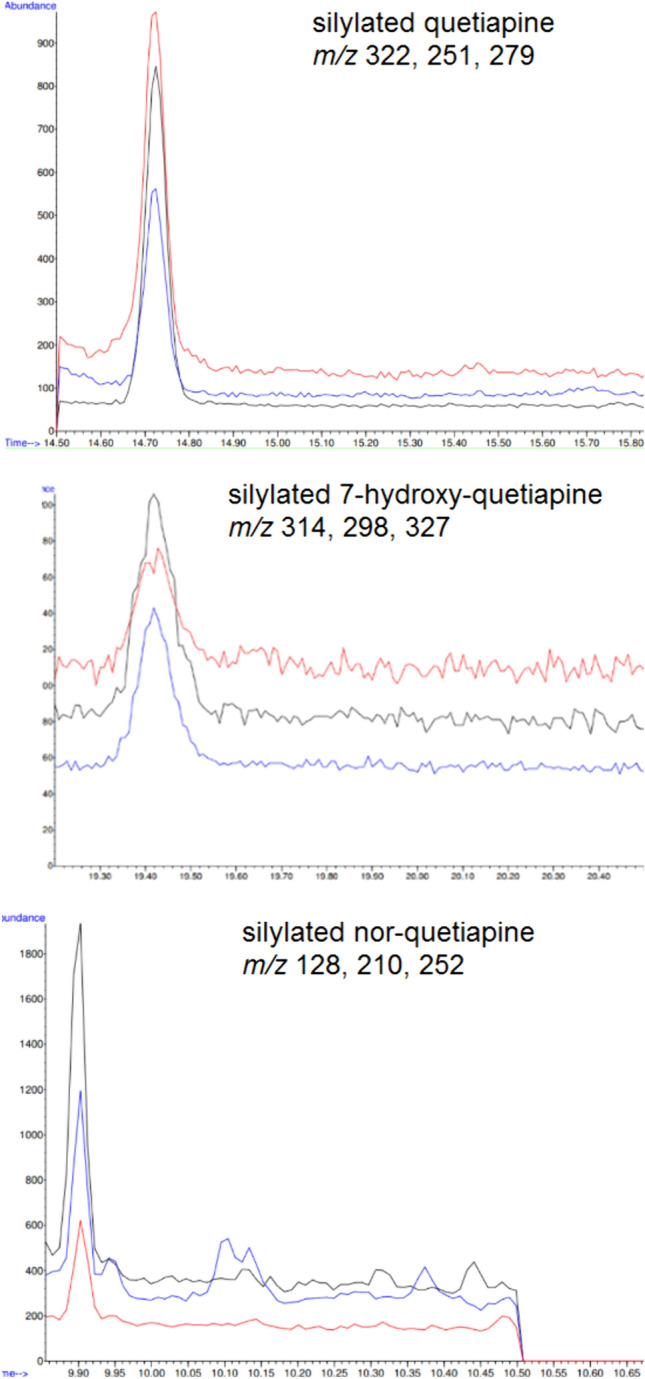
Representative selected ion monitoring chromatograms of a spiked vitreous humor sample with the three analytes of interest at the limit of quantification concentration (10.0 ng/mL)

In terms of sensitivity, the LOD and LOQ for all analytes of interest were found to be 3.00 and 10.0 ng/mL, respectively. SIM chromatograms of a spiked vitreous humor sample with all three analytes at the LOQ concentration (10.0 ng/mL) are shown in Fig. 2.

During linearity study, different calibration curves of the three analytes in blood and vitreous humor were calculated and the mean calibration curves are presented in Table 1. Furthermore, the coefficient of determination (*R*^*2*^) was calculated for each analyte and found to be higher than 0.993, 0.992, and 0.991 for quetiapine, 7-hydroxy-quetiapine, and nor-quetiapine, respectively. Furthermore, the % RSD of slopes was also calculated and was found to be less than 2.3, 3.3, and 4.6% for quetiapine, 7-hydroxy-quetiapine, and nor-quetiapine, respectively.

**Table 1:** The mean calibration curves of the three analytes in blood and vitreous humor

Analyte of interest	Blood	Vitreous humor
Quetiapine	*y* = 0.0294(± 0.0007)*x*–0.0449(± 0.0292)	*y* = 0.0304(± 0.0009)*x*–0.0513(± 0.0133)
7-Hydroxy-quetiapine	*y* = 0.0161(± 0.0005)*x*–0.1009(± 0.0215)	*y* = 0.0169(± 0.0007)*x*–0.1328(± 0.0255)
Nor-quetiapine	*y* = 0.0516(± 0.0024)*x*–0.3483(± 0.0150)	*y* = 0.0451(± 0.0037)*x*–0.4045(± 0.0112)

x: the concentration of each analyte

y: the peak area ratio

Intra-day precision was less than 5.3, 8.7, and 10.3% for quetiapine, 7-hydroxy-quetiapine, and nor-quetiapine, respectively; whereas, the corresponding inter-day precision values were found to be less than 4.3, 7.1 and 9.0%. Intra-day accuracy for quetiapine was found to be between −5.1 and 6.4%, for 7-hydroxy-quetiapine between −8.1 and 4.5%, and for nor-quetiapine between −6.5 and 3.4%. The inter-day accuracy was found to be between −2.2 and 1.2% for quetiapine, between −5.7 and 3.2% for 7-hydroxy-quetiapine, and between −1.4 and 2.6% for nor-quetiapine.

Absolute recovery for both biological samples was found to be higher than 81, 88, and 89% for quetiapine, 7-hydroxy-quetiapine, and nor-quetiapine, respectively, while matrix effect was found to be from 91 to 99% for quetiapine, from 93 to 103% for 7-hydroxy-quetiapine, and from 95 to 99% for nor-quetiapine, respectively.

Following its validation, the developed method was successfully applied to postmortem vitreous humor and blood samples obtained from 16 real forensic cases, where quetiapine was either detected in routine analysis or was included in the case history. Data of these cases are displayed in Table 2. Postmortem samples used were obtained from forensic cases investigated in the Department of Forensic Medicine and Toxicology of National and Kapodistrian University of Athens.

**Table 2:** Concentrations found in blood and vitreous humor samples of studied forensic cases

Cases	Blood concentration (ng/mL)			Vitreous humor concentration (ng/mL)			Vitreous humor /blood concentration ratio			Cause of death	Age
	Quetiapine	7-Hydroxy-quetiapine	Nor-quetiapine	Quetiapine	7-Hydroxy-quetiapine	Nor-quetiapine	Quetiapine	7-Hydroxy-quetiapine	Nor-quetiapine		
1	29.21	18.78	65.76	46.4	46.94	51.01	1.59	2.50	0.78	Suicide	63
2	55.87	32.81	66.03	72.1	74.8	231.3	1.29	2.28	3.50	Hanging	60
3	38.01	11.39	99.87	21.33	12.73	39.79	0.56	1.12	0.40	Undetermined	89
4	18.85	10.31	11.19	19.56	14.71	10.11	1.04	1.43	0.90	Undetermined	56
5	357.42	23.39	69.75	78.06	31.62	14.52	0.22	1.35	0.21	Drowning	46
6	10.41	10.25	139.55	26.45	21.13	87.94	2.54	2.06	0.63	Undetermined	85
7	529.26	89.04	164.99	280.08	32.22	15.98	0.53	0.36	0.10	Undetermined	50
8	60.20	10.70	< LOQ	17.9	20.58	18.41	0.30	1.92	–	Undetermined	61
9	31.64	13.42	51.09	13.94	12.03	11.71	0.44	0.90	0.23	Car accident	38
10	450.68	122.82	341.80	137.62	107.78	86.07	0.31	0.88	0.25	Undetermined	41
11	47.36	12.49	17.03	36.36	17.02	60.66	0.77	1.36	3.56	Heat burns	55
12	106.65	17.84	33.62	22.54	30.30	34.23	0.21	1.70	1.02	Undetermined	58
13	4187.61	161.99	20.28	289.40	171.8	254.57	0.07	1.06	12.55	Undetermined	47
14	74.84	16.8	104.81	129.47	88.05	301.06	1.73	5.24	2.87	Fall from a height	64
15	10.89	10.30	14.41	10.96	22.05	17.26	1.01	2.14	1.20	Undetermined	94
16	1740.86	64.84	1132.82	98.57	26.89	119.85	0.06	0.41	0.11	Undetermined	59

Quetiapine, 7-hydroxy-quetiapine, and nor-quetiapine were quantified in both matrices using the respective calibration curves constructed at the day of the analysis. Moreover, to interpret the distribution of the analytes in vitreous humor, the concentration ratios of the analytes in vitreous humor to the respective in blood were calculated. Quantification results for quetiapine, 7-hydroxy-quetiapine, and nor-quetiapine were obtained, along with the concentration ratios of vitreous humor and blood samples and are shown in Table 2.

The mean values of blood and vitreous humor concentration for quetiapine, 7-hydroxy-quetiapine, and nor-quetiapine, as well as the mean ratios of vitreous humor to respective blood concentrations are presented in Table 3. For more solid conclusions, two cases (cases 13 and 16) where quetiapine’s concentration in blood was toxic (above 1000 ng/mL) were excluded from these calculations.

**Table 3:** Mean values of blood, vitreous humor concentrations, and vitreous humor/blood concentration ratios of the studied cases

Analyte	Mean blood concentration (ng/mL)	Mean vitreous humor concentration (ng/mL)	Mean vitreous humor/ blood concentration ratio
Quetiapine	130.10	65.20	0.89
7-Hydroxy-quetiapine	28.60	38.00	1.80
Nor-quetiapine	90.76	70.00	1.20

## Discussion

According to the results obtained, it was found that when a case was positive for quetiapine and/or its metabolites in blood, the substances were also detected in vitreous humor. This suggests that the three studied substances are well distributed in vitreous humor.

The mean concentration of quetiapine in postmortem blood samples was 130.10 ng/mL, while in vitreous humor was 65.20 ng/mL. The ratio of the concentrations of each analyte in vitreous humor to blood was calculated and ranged from 0.02 to 2.54, with a mean value of 0.89. This significant variation of the ratios is explained by the fact that the time interval between the drug’s intake and death varies. In cases where vitreous humor to blood ratio is notably higher than the mean ratio (cases 1, 6, and 14), it is indicated that the above-mentioned interval could be long. On the contrary, when a much lower concentration ratio than the mean value is observed, it may be due to drug overdose, possible postmortem redistribution, or a short intake-to-death interval. In cases where postmortem blood concentration of quetiapine is at toxic levels, the distinguishing of drug overdose and postmortem redistribution is of great interest.

The mean concentration of 7-hydroxy-quetiapine was 28.60 and 38.00 ng/mL in blood and vitreous humor samples, respectively. The concentration ratio of this metabolite was found to be between 0.36 and 5.24, with mean value of 1.80. 7-hydroxy-quetiapine’s relatively higher polarity than quetiapine increases its solubility in vitreous humor, which consists of water by 99%, justifying the higher ratios of this metabolite, compared to the parent drug.

As for nor-quetiapine, it was observed that this metabolite may be detected in vitreous humor at a higher concentration than quetiapine. The mean concentration of nor-quetiapine in postmortem blood samples was 90.76 ng/mL, and in vitreous humor 70.00 ng/mL, whereas the concentration ratios were found to be between 0.10 and 12.55, with a mean value of 1.20.

The developed and validated method for all three substances, displayed increased sensitivity compared to the published method of Flammia et al. [21], which was determining solely quetiapine in blood, vitreous humor, and other specimens (50 ng/mL), as well as the studies of Rosado et al. [30] and Lopez- Guarnido et al. [26] which were analyzing only quetiapine in blood (10 ng/mL). The present method has been validated for a wider dynamic range of 10.0–1000.0 ng/mL for vitreous humor and blood, as opposed to other published methods by Rosado et al. (40–600 ng/mL) [30], Caramelo et al. (10–400 ng/mL) [31], and Lopez-Guarnido et al. (20–1000 ng/mL) [26]. To our knowledge, the present method is the only GC–MS method that simultaneously identifies quetiapine along with its two metabolites (7-hydroxy-quetiapine and nor-quetiapine) in blood and in vitreous humor.

The observed significant distribution of quetiapine and its two main metabolites to the vitreous humor compartment indicates the importance of this matrix as an alternative biological specimen in forensic toxicology for the qualitative determination of these compounds, even in cases when classical biological specimens, such as blood and urine, are absent or the sample’s collected volume is not the requisite for analysis. To our knowledge, this work is the first to study the distribution of quetiapine’s metabolites in vitreous humor.

From the results obtained, in two cases quetiapine was found at toxic levels and are further discussed. One of the two cases (case 13) was confirmed as a quetiapine overdose, based on its history with a blood concentration of quetiapine of 4187.61 ng/mL and a vitreous humor concentration of 289.40 ng/mL, notably higher than the calculated average concentration (65.20 ng/mL) during this study. However, this observation contradicts the proposed limit (500 ng/mL) set by Parker et al. [20] to differentiate quetiapine’s therapeutic and toxic concentrations in vitreous humor. The fact that similar concentration of quetiapine in vitreous humor was found in a case where the concentration of quetiapine in blood was considered therapeutic (case 7280.08 ng/mL), suggests that the concentration of quetiapine in vitreous humor cannot be used as the only evidence to investigate its role in the cause of death. In case 13, the quantification of 7-hydroxy-quetiapine in vitreous humor at a concentration of 171.80 ng/mL, critically higher than the average (38.00 ng/mL), may suggest that the simultaneous determination of quetiapine and 7-hydroxy-quetiapine in vitreous humor may be more helpful than that of solely quetiapine for the investigation of quetiapine-related deaths. Moreover, it has been observed that in case 16, where the concentration of quetiapine in blood was toxic, quetiapine and 7-hydroxy-quetiapine in vitreous humor were found at average level concentrations. This could suggest that the detection of quetiapine and/or 7-hydroxy-quetiapine in vitreous humor at a concentrations equal or lower than their respective mean value might exclude the drug from being linked to the cause of death, even when quetiapine’s concentration in blood is determined as toxic. Considering the above, in case 16, it is believed that the high quetiapine concentration found in blood is most likely a result of its postmortem redistribution than its ante mortem overdose. From the analysis of the results it was concluded that the simultaneous determination of both quetiapine and 7-hydroxy-quetiapine in vitreous humor at higher (≥ 250 ng/mL for quetiapine and ≥ 150 ng/mL for 7-hydroxy-quetiapine) than mean levels is likely to support the intake of large amounts of quetiapine and its possible association with the cause of death. For nor-quetiapine, despite the fact that it was always detected in vitreous humor samples, no correlation with the respective blood concentrations was found.

For more solid conclusions, it is necessary to examine more quetiapine-related cases, especially cases with a clear history of quetiapine overdose, to determine therapeutic and toxic concentrations of quetiapine and its metabolites in vitreous humor. This will establish vitreous humor as an important alternative material in forensic toxicology for the determination of quetiapine and its metabolites.

## Conclusion

Alternative postmortem matrices, such as vitreous humor, are of significant value in investigating forensic cases due to their analytical advantages, but their utility in toxicological analysis requires progressive research. This is the first study to investigate the distribution of both quetiapine and its metabolites in vitreous humor. According to the results of the present study, quetiapine, 7-hydroxy-quetiapine, and nor-quetiapine are easily distributed in vitreous humor. This suggests the usefulness of vitreous humor in toxicological analysis for the qualitative determination of these compounds when the classical biological materials are not available. In addition, the quantification of both quetiapine and its metabolite 7-hydroxy-quetiapine in vitreous humor can provide significant information for a more accurate investigation of forensic cases. Hence, more studies are needed for the determination of therapeutic and toxic concentrations of quetiapine and its metabolites in this matrix is of great importance.
